# *exampletestr*—An easy start to unit testing R packages

**DOI:** 10.12688/wellcomeopenres.11635.2

**Published:** 2017-06-21

**Authors:** Rory Nolan, Sergi Padilla-Parra

**Affiliations:** 1 Wellcome Trust Centre for Human Genetics, University of Oxford, Oxford, OX3 7BN, UK; 2 Department of Structural Biology, University of Oxford, Oxford, OX3 7BN, UK

**Keywords:** R, unit, test, testing, testthat, covr, exampletestr

## Abstract

In spite of the utility of unit tests, most R package developers do not write them. *exampletestr *makes it easier to *start *writing unit tests by creating shells/skeletons of unit tests based on the examples in the user's package documentation. When completed, these unit tests test whether said examples run *correctly*. By combining the functionality of *exampletestr *with that of *covr*, having ensured that their examples adequately demonstrate the features of their package, the developer can have much of the work of constructing a comprehensive set of unit tests done for them.

## Introduction

Unit tests are automated checks which examine whether a package works as *intended*. There are many reasons to write unit tests:

1. **To identify bugs in your current code.** If a test fails, something is not working as expected.
2. **To make current package functionality robust to future changes.** If you have written good tests, you do not need to worry that changes made to your code have broken existing functionality; the tests will tell you whether or not the original functionality is in tact. In the words of Hadley Wickham, "I started using automated tests because I discovered I was spending too much time refixing bugs that I’d already fixed before." ^1^
3. **Test-driven development (TDD).** Some people advocate writing tests before writing code: the tests outline what your code should do and you know your code is working correctly when the tests start passing. The use of TDD achieves the quality control goals of points 1 and 2 above as code is written. This contrasts with *retrospective unit testing*, which is to write unit tests after everything else is done.
4. **To assure others that your code works correctly.** The fact that a package is unit tested indicates to potential users that the author has taken care to ensure that their code works as intended.

For the above reasons, it is good practice to write unit tests for R packages. However, at the time of writing, of the 10084 packages on CRAN, only 25% (2566) are unit tested. Of the 5715 packages authored or updated since 1st January 2016, 35% (1984) are unit tested. Hence, the majority of packages on CRAN are not unit tested. Testing for these packages would have to be done retrospectively.

This paper describes exampletestr, a package that makes it easy for package developers to retrospectively create unit tests based on the examples in their package documentation. exampletestr is designed for developers who have a functioning package that they would like to unit test; it is less useful for those using TDD.

## Methods

### Implementation

exampletestr is designed for tests to be written within the testthat ^2^ framework. This is the most popular testing framework, preferred in 93% of cases. exampletestr works by reading the examples in the .Rd files in the man/ folder of the package project. These examples are then wrapped in test_that and expect_equal statements, thereby creating *test shells*.

By running make_tests_shells_pkg() in the root directory of a package, for each x.R file in the R/ directory that has at least one function documented with an example, you get a corresponding file test_x.R in the tests/testthat/ directory of the package, containing these test shells to be filled in by the user (a package developer) to create fully functional unit tests.

For a complete overview of exampletestr’s capabilities, consult the package’s manual and vignette at https://CRAN.R-project.org/package=exampletestr.

### Operation

This package can be used with R version 3.3.0 or later on Linux, Mac and Windows. It can be installed from within R via the command install.packages("exampletestr").

The idea of basing unit tests around documentation examples suggests the use of covr ^3^ in the following way to ensure both that the examples in the documentation exhibit as much of the functionality of the package as possible and that the unit tests cover as much of the code as feasible:

1. Write comprehensive documentation examples for your package, using covr’s package_coverage(type = "examples") %>% shine() to ensure that all sections of code that the package user may find useful are demonstrated.
2. Write unit tests corresponding to *all* of your examples using exampletestr.
3. Complete the writing of unit tests, checking your code coverage with package_coverage(type = "tests") %>% shine().
Using this workflow, the developer ensures that their *example coverage* (the portion of package features covered by documentation examples) is adequate, and simultaneously obtains a reduction in the work required to write comprehensive unit tests.

## Use cases

The best way to display the functionality of exampletestr is by example. Take the str_detect function from the stringr package ^4^. The main file str_detect.Rd has the examples section:



                    \examples{
fruit <- c("apple", "banana", "pear", "pinapple")
str_detect(fruit, "a")
str_detect(fruit, "^a")
str_detect(fruit, "a$")
str_detect(fruit, "b")
str_detect(fruit, "[aeiou]")
# Also vectorised over pattern
str_detect("aecfg", letters)
}
                


The *test shell* that would be automatically created by exampletestr from these examples is:



                    test_that("str_detect works", {
  fruit <- c("apple", "banana", "pear", "pinapple")
  expect_equal(str_detect(fruit, "a"), )
  expect_equal(str_detect(fruit, "^a"), )
  expect_equal(str_detect(fruit, "a$"), )
  expect_equal(str_detect(fruit, "b"), )
  expect_equal(str_detect(fruit, "[aeiou]"), )
  expect_equal(str_detect("aecfg", letters), )
})
                


which can then be filled in by the package developer to give the complete and passing test:




                    test_that("str_detect works", {
  fruit <- c("apple", "banana", "pear", "pinapple")
  expect_equal(str_detect(fruit, "a"), rep(TRUE, 4))
  expect_equal(str_detect(fruit, "^a"), c(TRUE, rep(FALSE, 3)))
  expect_equal(str_detect(fruit, "a$"), c(FALSE, TRUE, FALSE, FALSE))
  expect_equal(str_detect(fruit, "b"), c(FALSE, TRUE, FALSE, FALSE))
  expect_equal(str_detect(fruit, "[aeiou]"), rep(TRUE, 4))
  expect_equal(str_detect("aecfg", letters), 
                c(TRUE, FALSE, TRUE, FALSE, TRUE, TRUE, TRUE, rep(FALSE, 19)))
})



## Discussion

Testing whether documentation examples run correctly (which is what exampletestr is useful for) is just one important part of writing good unit tests. Two points to bear in mind when using exampletestr:

1. Documentation examples typically showcase a *best case* application of the code in a package. Unit testing should go beyond this and assess the execution of edge cases, e.g. the performance of functions when an argument is empty (length zero). exampletestr does not help in this regard.
2. exampletestr promotes a “one test per function” model. However, in many packages, it is necessary to go further than just testing whether each function performs well in isolation. For instance, it is good to test function composition: whether one obtains the expected results when applying two or more functions, one after another.

## Conclusions

Unit tests are crucial to ensuring that a package functions correctly, yet most developers do not write them. Most package developers do, however, write documentation examples. With exampletestr, documentation examples are easily transformed into unit tests; thereby encouraging maintainers of untested packages to make a start on unit testing.

## Software and data availability

exampletestr can be downloaded from: https://CRAN.R-project.org/package=exampletestr

Source code available from: https://github.com/rorynolan/exampletestr

Archived source code as at time of publication: DOI: 10.5281/zenodo.575664

Software license: GPL-3

The data presented can be found at https://github.com/rorynolan/exampletestr/tree/master/analysis, along with a file showing how to reproduce that data.
