# Supplementary material for: exampletestr—An easy start to unit testing R packages
Source: Wellcome Open Res. 2017 Jun 21;2:31. Originally published 2017 May 17. [Version 2] doi: 10.12688/wellcomeopenres.11635.2 (PMC5500893; doi:10.12688/wellcomeopenres.11635.2)
Supplement: Peer review history

# Peer review: Referee response for version 2

**DOI:** 10.21956/wellcomeopenres.12933.r23685

**Published:** 2017-06-29

**Authors:** Laurent Gatto

**Affiliations:** 1 Computational Proteomics Unit, University of Cambridge, Cambridge, UK

Thank you for the amendments, Rory. I am happy to accept the publication.

I have read this submission. I believe that I have an appropriate level of expertise to confirm that it is of an acceptable scientific standard.

# Peer review: Referee response for version 1

**DOI:** 10.21956/wellcomeopenres.12567.r23062

**Published:** 2017-06-12

**Authors:** Laurent Gatto

**Affiliations:** 1 Computational Proteomics Unit, University of Cambridge, Cambridge, UK

The manuscript *Easier unit tests and better examples with exampletestr and covr* describes the `exampletestr` package that parses the example code in the manual pages and converts it into unit test stubs to be then completed by the developer.

 I read the manuscript and found it was clearly written. I also successfully executed the examples in the package vignette. In general, I believe the authors have prepared a useful package and have described it well.

 I have however two important points I would like to raise.

 My first point is that I think it would be useful for the authors to provide a better rational for unit testing. In my opinion, the current version of the text is obvious to those that already know about and practice unit testing. However, the goal of this work is to appeal to the many that haven't adopted unit testing in their standard development yet. For those, it might be useful to provide more background and to emphasise the important of unit testing.

 My second point is that by focusing on code from examples, some of the most important unit tests are systematically missed out. Indeed, one generally documents a typical, default, *best case* application of the code/package in the documentation files. One could argue that the goal of unit testing is to go beyond these, and assess the execution of the edge cases. Using some material from my own work, some example would be

 - to test the validity of empty data/objects (where users wouldn't, generally, produce empty data instances, and this wouldn't be described in the manual page);

 - verify that an example object that is distributed with the package (and loaded with `load`), that was created from a distributed example files (for instance in `inst/extdata/`), remained valid and identical throughout the evolution of the classes and constructors;

 - other examples illustrate how unit tests can be build and refined against a set of functions specifications, i.e. expected outputs.

 Similarly, code presented in examples illustrate exported and high level code, while unit tests might rather want to address specific and private functions, that in turn are called within the high level user space functions.

 Another important point is that writing (or thinking about) unit tests often influences how one writes code, testable code, which is not reflected in writing code in the manual examples.

 Also, although efficiency (in terms of time when checking the package) is not a concern, at least in a first instance, a side effect of basing unit test on examples, results in re-running the same code twice. For unit testing, it might be worth wile to extend code

 coverage to rarely used lines of code by tuning arguments accordingly.

 In the vignette, the author clearly states that "The Goal is NOT Fully Automated Unit Test Creation". May be this could also be mentioned in the manuscript.

 A minor suggestion would be to drop `covr` from the title, as it might mislead readers that the manuscript also addresses directly that package.

 Also, I was wondering what happened if an example code chunk was not run (wrapped in `\dontrun`). Is it ignores by `exampletestr`?

 To conclude, I think the manuscript would benefit greatly from a better introduction/discussion for newcomers. The second issue I have relates to the scope or validity of basing unit test on examples. While I do not expect `exampletestr` (or any other package) to come up with valid general unit tests generation recipe, I think it

 would be essential to recast and discuss unit testing into a broader scope.

I have read this submission. I believe that I have an appropriate level of expertise to confirm that it is of an acceptable scientific standard, however I have significant reservations, as outlined above.

# Response

**Published:** 2017-06-13

**Authors:** Rory Nolan

Hi Laurent,

 Thanks for the review and advice. I agree on all points and I'll edit the manuscript accordingly. I'll submit my revisions within the next 2 weeks. 

 For now, to answer your question, \dontrun{} blocks are ignored. I considered putting them into expect_error() statements but decided against it. I thought there was no good way to systematically deal with them. If you have a suggestion, please let me know.

 I've edited the package documentation to make it clearer what happens to \dontrun{} blocks.

 Thanks again,

 Rory

# Response

**Published:** 2017-06-16

**Authors:** Rory Nolan

Dear Laurent,

 I have submitted a new version of the manuscript. I hereby outline my response to your comments.

**I think it would be useful for the authors to provide a better rational for unit testing. In my opinion, the current version of the text is obvious to those that already know about and practice unit testing. However, the goal of this work is to appeal to the many that haven't adopted unit testing in their standard development yet. For those, it might be useful to provide more background and to emphasise the important of unit testing.**

 I have done this now in the introduction.

**By focusing on code from examples, some of the most important unit tests are systematically missed out. Indeed, one generally documents a typical, default, best case application of the code/package in the documentation files. One could argue that the goal of unit testing is to go beyond these, and assess the execution of the edge cases.**

 I have included this point in the discussion.

**Writing (or thinking about) unit tests often influences how one writes code, testable code, which is not reflected in writing code in the manual examples.**

 This is a good point. exampletestr is made for packages in which the author did not write tests as the package was being developed. In the manuscript I now try to make it clear that this isn't necessarily a good way to develop a tested package, but that it is the way that most packages are developed and hence exampletestr is a useful tool to begin testing these packages.

**Although efficiency (in terms of time when checking the package) is not a concern, at least in a first instance, a side effect of basing unit test on examples, results in re-running the same code twice.**

 This is unfortunate, however I think having a set of unit tests which affirm that your examples are working properly very useful and worth having in spite of this re-running (which is, as you eluded to, not a big concern most of the time).

**For unit testing, it might be worth wile to extend code coverage to rarely used lines of code by tuning arguments accordingly.**

 This is an important point. I think this is taken care of by the workflow that makes use of covr.

**In the vignette, the author clearly states that "The Goal is NOT Fully Automated Unit Test Creation". May be this could also be mentioned in the manuscript.**

 I decided not to copy this in explicitly because I think the discussion now makes clear the point that there is much more to unit testing than what exampletestr can do for you.

**A minor suggestion would be to drop `covr` from the title, as it might mislead readers that the manuscript also addresses directly that package.**

 I have done this. Please let me know if you think the new title could be improved.

**Also, I was wondering what happened if an example code chunk was not run (wrapped in `\dontrun`). Is it ignores by `exampletestr`?**

 \dontrun{} blocks are ignored. I considered putting them into expect_error() statements but decided against it. I thought there was no good way to systematically deal with them. If you have a suggestion, please let me know.

**To conclude, I think the manuscript would benefit greatly from a better introduction/discussion for newcomers. The second issue I have relates to the scope or validity of basing unit test on examples. While I do not expect `exampletestr` (or any other package) to come up with valid general unit tests generation recipe, I think it would be essential to recast and discuss unit testing into a broader scope.**

 I have tried to address all of this. Thank you for your insightful review.

 Rory

# Peer review: Referee response for version 1

**DOI:** 10.21956/wellcomeopenres.12567.r22849

**Published:** 2017-05-30

**Authors:** Thomas Quinn

**Affiliations:** 1 Bioinformatics Core Research Group, Deakin University, Geelong, Vic, Australia

The authors clearly outline the importance of unit testing. They then introduce a new package aimed to make it easier for developers to add unit tests to their own package. They do this by drawing from examples provided in the .Rd documentation. As such, the exampletestr package is automatically compatible with Roxygen2. Importantly, the authors outline unit tests using the testthat package, the standard unit testing package. These features make exampletestr consistent with best practices for R package development.

 I used exampletestr::make_tests_shells_pkg() on an old deprecated package that I am renovating for the newest version of R. At first, I encountered an error; however, the package provided precise instructions on how to resolve the error. Otherwise, the exampletestr package works exactly as advertised. In my opinion, exampletestr would work nicely in combination with the Rd2roxygen package to help bring antiquated R packages up to a more rigorous and maintainable software standard.

I have read this submission. I believe that I have an appropriate level of expertise to confirm that it is of an acceptable scientific standard.

# Response

**Published:** 2017-06-16

**Authors:** Rory Nolan

Dear Thomas,

 I have submitted a revised version of the article to address Laurent Gatto's review.

 This new version puts the package into better context.

 The package has been updated such that the error that you got doesn't happen any more.

 Thanks,

 Rory
